# The Usefulness of Combining Noninvasive Methods for Early Identification and Potential Prevention of Cystic Fibrosis-Associated Liver Disease

**DOI:** 10.7759/cureus.32340

**Published:** 2022-12-09

**Authors:** Twisha S Shukla, Sai Dheeraj Gutlapalli, Hadi Farhat, Huma Irfan, Kanmani Muthiah, Namratha Pallipamu, Sogand Taheri, Suvedha S Thiagaraj, Pousette Hamid

**Affiliations:** 1 Pediatrics, California Institute of Behavioral Neurosciences & Psychology, Fairfield, USA; 2 Internal Medicine, California Institute of Behavioral Neurosciences & Psychology, Fairfield, USA; 3 Cardiology, Rheumatology, California Institute of Behavioral Neurosciences & Psychology, Fairfield, USA; 4 Cardiology, Rheumatology, University of Balamand, Beirut, LBN; 5 Research, California Institute of Behavioral Neurosciences & Psychology, Fairfield, USA; 6 Neurology, California Institute of Behavioral Neurosciences & Psychology, Fairfield, USA; 7 Medicine, California Institute of Behavioral Neurosciences & Psychology, Fairfield, USA

**Keywords:** pediatrics, cystic fibrosis complication, prevention, liver and gall bladder disease, cystic fibrosis

## Abstract

Cystic fibrosis-associated liver disease is the third leading cause of morbidity and mortality in patients with cystic fibrosis (CF). Liver damage in the course of CF ranges from biochemical abnormalities to full-blown cirrhosis and may involve complicated processes like inflammation, fibrogenesis, remodeling, apoptosis, and cholestasis. Despite robust research in the field of CF, its complex pathogenesis is not fully understood. Because of the unknown pathogenesis, it is difficult to develop a highly sensitive and specific test or technology that is standardized, acceptable, and available at most pediatric institutions. The Cystic Fibrosis Foundation (CFF) recommends annual blood tests to screen for liver pathology, which often fails to identify early-onset liver disease. In this review article, we present the use of different liver indices and imaging modalities that can help identify liver disease at the onset and may help in its prevention. Although the disease is commonly diagnosed in the pediatric population, due to increased life expectancy, there is increasing evidence of liver disease in adults too. We believe that the tools we present in this review will help in the prevention of liver disease and thereby reduce the associated morbidity and mortality.

## Introduction and background

Cystic fibrosis (CF) is caused by a mutation of the cystic fibrosis transmembrane regulator (CFTR) gene on the long arm of chromosome 7 [1], which encodes the CFTR protein. It is an autosomal-recessive disorder that most commonly affects Caucasians. In the United States, the incidence of CF is 1/4,000 live births [2].

Liver disease (LD) in CF is a leading cause of morbidity and mortality. Complications from liver diseases are the third leading cause of death in CF after lung complications and transplant complications. According to the Cystic Fibrosis Foundation (CFF), liver disease in CF can range from neonatal cholestasis to multinodular cirrhosis. Based on the currently available clinical, biochemical, and radiological data, the prevalence of LD is estimated at 26-45% [3]. However, these prevalence rates are likely an underestimation since in older autopsy studies focal biliary cirrhosis is reported in 72% of the cases [4]. Up to 15% develop severe liver abnormalities, and up to 5% require a liver transplant or die due to end-stage liver disease or cirrhosis [5,6]. Liver biopsy is considered the gold standard for assessing stages of fibrosis but is known to be unreliable due to the focal nature of cystic fibrosis liver disease (CFLD) [7].

As there is no universally accepted definition of CFLD, it is challenging to devise new screening tools to identify the early onset of the disease. Identifying children at risk of cirrhosis and halting the progression to portal hypertension and liver failure is critical. As fibrosis is an intermediary step in the development of cirrhosis, detecting and monitoring fibrosis would enhance the study of potential interventional therapies. Therefore, early diagnosis to identify trends and prompt intervention are necessary to improve treatment plans and quality of life. A sensitive, specific, and noninvasive screening process that helps to identify the early pathogenesis of CFLD is required to identify individuals at risk of developing hepatic fibrosis before the advent of severe complications [8].

Since CFLD develops early in life and new cases after 20 years of age are rare [1,2], those in the pediatric age group are the most pertinent population for screening and diagnosing CFLD. The objective of this review is to identify early markers of liver disease in CF, which can be used in screening and planning the management of the condition. Early diagnosis enables timely initiation of ursodeoxycholic acid therapy and monitoring of the evolution of CFLD [3]. We believe that the findings of this review can help to devise a combination of methods that can aid in the diagnosis and early identification of liver disease, which can help prevent adverse outcomes.

## Review

Liver cirrhosis is a well-recognized complication of CF and causes significant morbidity and mortality in CF patients [8]. With cystic fibrosis transmembrane conductance regulator (CFTR) expressed in the biliary epithelium, not in hepatocytes, the earliest marker of liver fibrosis is likely to arise from the bile ducts. Mutation in the CFTR causes impaired intrahepatic biliary ductal secretion resulting in inspissated bile and bile duct plugging, which leads to inflammation, liver injury, and biliary fibrosis [9]. However, there is a wide spectrum of liver pathology associated with CF, including steatosis, fibrosis, and focal or multinodular cirrhosis.

CFF and European liver disease guidelines recommend annual assessment through liver blood tests [serum aspartate aminotransferase (AST), alanine aminotransferase (ALT), gamma-glutamyl transferase (GGT), bilirubin, and alkaline phosphatase] in all CF patients as part of screening for liver disease. Due to the unknown pathogenesis of liver disease and cirrhosis in CF patients, labs alone are not sufficient and often fail to identify mild diseases. With 90% of end-stage liver disease cases occurring in childhood, liver cirrhosis is generally considered a pediatric complication of CF [10]. Classic noninvasive assessments of liver fibrosis are not validated for the pediatric population and several CF-associated processes can cloud the diagnosis (e.g., infection and medication can modify classic noninvasive markers of liver disease such as AST). The most widely accepted diagnostic criteria for CFLD are the EuroCare CFLD criteria, which include more than two of the following manifestations: persistent abnormal liver biochemistry for 12 months, hepatomegaly and/or splenomegaly, or ultrasound abnormalities [11].

Liver biopsy is currently the gold standard for assessment for liver cirrhosis but is an invasive procedure subject to inter-observer variability and sampling error of biopsies. There is a number of other complications like discomfort, sedation complications [12], bleeding, pneumothorax, and infective peritonitis which limits the use of liver biopsies in all patients [13]. Moreover, only 1/50,000 of the liver volume is investigated resulting in sampling error in focally distributed liver disease as CFLD [14]. The ultrasound-guided biopsy can be used to avoid under or over-estimation of disease. Some studies suggest that only during the early stages are histopathological changes reversible and may be efficaciously treated [15].

There are other widely accepted studies that are validated in other liver conditions and have been studied to some extent in cystic fibrosis liver diseases described here.

The Fibrosis-4 (FIB-4) index

$FIB-4 = \frac{Age(years) \times AST[U/L]}{Platelets[10^9/L] \times (\sqrt{ALT}[U/L] )}$

The Fibrosis-4 (FIB-4) index is a noninvasive test for the assessment of liver fibrosis, which has been studied widely in hepatitis C and nonalcoholic steatohepatitis (NASH) but not in CF. A score <1.45 and >3.25 enables the correct identification of adult patients who have moderate or significant fibrosis in hepatitis C [16]. Another study done in children with biopsy-proven nonalcoholic fatty liver disease (NAFLD) showed poor diagnostic accuracy for fibrosis and concluded that noninvasive hepatic fibrosis scores developed in adults had poor performance in diagnosing fibrosis in children with NAFLD [17].

In multiple studies, the FIB-4 index has shown poor diagnosis quality in predicting liver disease [18,19] with a calculated area under the receiver operating characteristic (AUROC) of 0.656 and 95% CI of 0.511-0.801 compared with the liver stiffness measurement (LSM) test [19]. This AUROC value is similar to what Daniel et al. found in their study comparing CFLD and cystic fibrosis without liver disease (CFnoLD) [12].

Aspartate aminotransferase-to-platelet ratio (APRI)

AST-to-platelet ratio (APRI) is a cost-effective blood test-based scoring system that can predict liver fibrosis. It has been studied in children infected with hepatitis B and C [20,21] and biliary atresia [22,23]. APRI is calculated as follows:

$APRI = \frac{\frac{AST Level(IU/L)}{ULN of AST}}{Platelet counts(10^9/L)} \times 100$

A retrospective, cross-sectional study by Daniel et al. identified that the area under the curve (AUC) for APRI in detecting liver disease was significantly better than the FIB-4 index. Based on Youden’s index [summary of the receiver operative characteristic curve (ROC) curve], an APRI score >0.264 demonstrated a sensitivity of 70.2% [12]. A 50% increase in APRI score among CF patients is associated with a 2.4-fold increased odds of having CFLD. This study was able to distinguish no fibrosis (F0) from CFnoLD with median APRI scores that were higher in F0. With the help of the logistic regression model, APRI was significantly associated with the fibrosis stage. A one-unit increase in the APRI score is associated with a 3.2-fold increased odds of advancing to the next stage. But during this study, APRI overestimated fibrosis stage 45% of the time and underestimated fibrosis 18% of the time.

Variability in the upper limit of normal values used in these calculations makes it difficult to compare results across research studies and identify appropriate indices cut-offs [24]. In the July issue of the Journal of Cystic Fibrosis, Karnsakul et al. [18] examined the efficacy of noninvasive biomarkers to identify and monitor the progression of CFLD in pediatric CF patients and calculated a cut-off value of APRI of >0.437 and gamma-glutamyl transpeptidase (GGT)-to-platelet ratio (GPR) of >0.281. Sellers et al. came up with the cut-off of >0.367 and >0.682 for APRI and GPR respectively. This difference in cut-offs is mainly due to the difference in the upper limit of the normal value of AST and GGT. The study done by Sellers et al. based on the standardized cut-off by Canadian Laboratory Initiative on Pediatric Reference Intervals (CALIPER) Project ULN values (AST: 25-46, GGT: 16 or 21, based on age/sex) helped mitigate the difference between cut-off values. This study’s cut-offs were closer to the previous ones with APRI of >0.433 and GPR of >0.198 [24]. APRI can be used as a highly sensitive test if the upper limit of normal values is standardized.

Gamma-glutamyl transpeptidase-to-platelet ratio (GPR)

GPR can be used for evaluating the grade of hepatic fibrosis [25,26] and has been noted to have higher sensitivity than APRI and FIB-4 [25] in cases of chronic hepatitis B.

Diego et al. assessed the usefulness of GPR as a noninvasive biomarker to evaluate for the presence of liver disease in children with CF [5]. The study showed that GPR was significantly elevated in CFLD vs. CFnoLD [0.33 (0.19-0.96) vs. 0.15 (0.11-0.21), p<0.0001]. A cut-off GPR value of 0.20 demonstrated optimal sensitivity of 74% and specificity of 73% with a positive predictive value (PPV) of 57% and a negative predictive value (NPV) of 85% [5]. Furthermore, GPR can also be used for distinguishing the severity of the liver disease. In this study, patients with mild/moderate/severe/advanced fibrosis (F1/F2/F3/F4) showed higher median GPR levels in comparison to patients with no fibrosis (F0) [5].

GPR scores are calculated as follows:

$GPR = \frac{\frac{(GGT level(IU/L)}{ULN of GGT}}{Platelet count(10^9/L)} \times 100$

GPR is more accurate, sensitive, and easy to use than the FIB-4 index and APRI, but when combined with FIB-4 and APRI, the sensitivity and specificity of diagnosis are significantly improved [27] In CFLD, focal biliary cirrhosis occurs before the onset of multi-lobular cirrhosis. As CFTR is expressed in the biliary epithelium, not in hepatocytes, the first marker arises from the biliary epithelium. Seller et al. revealed that the ideal cut-off for GGT is 21 U/L to differentiate between nodular and non-nodular liver disease, with an AUROC of 0.87 [10]. As GPR uses GGT values in the calculation, it indicates superiority over APRI and FIB-4.

Ultrasound

Ultrasonography is a frequently utilized tool for the diagnosis of liver disease in clinical research as well as care settings. CFF recommends the use of ultrasound (US) for children who are at risk of liver disease or those who have abnormal biochemical or clinical findings suggestive of liver disease. Among those in whom the US detects abnormal liver parenchyma, only a few progress to severe liver disease [28,29]. Although there has been a historically poor correlation between US findings and liver disease severity, a heterogeneous liver pattern on ultrasound is identified to be a potential marker for patients with CF who would later develop cirrhosis [29]. The importance of homogenous patterns is also unknown and yet to be studied; as it is a more common finding among obese children and NASH, it is often assumed to be due to hepatic steatosis in CF.

Cystic Fibrosis Liver Disease Network (CFLD-NET) is undertaking the PUSH study (Prediction by Ultrasound of the Risk of Hepatic Cirrhosis in Cystic Fibrosis) to identify at-risk children for severe liver disease, which could enable preventative therapies. The detailed methodology reported [30] was used by Simon et al. to identify the association of different biomarker indices including platelet counts, and spleen size, with ultrasound patterns of liver cirrhosis. They classified US findings by liver parenchymal pattern as normal (NL), heterogeneous (HTG), homogeneously hyperechoic (HMG), and nodular (NOD) [30]. Ultrasound has the advantage that it can detect more abdominal abnormalities other than just liver fibrosis, although the significance of these occasional findings is unclear.

The study was able to identify that ALT, AST, GGT, APRI, and FIB-4 were lower in ultrasonographic normal liver than NOD. When comparing NOD vs. NL, HTG vs. NL, NOD vs. HTG, and HMG vs. NL, significant associations were found. APRI, GGTP, ALT, and AST were statistically significant in all four models comparing the US. AUROC confirmed the excellent discriminating ability for NOD vs. NL when compared with GGT and APRI. But when comparing HTG vs. NL with GGT and APRI, it was fairly distinguished with AUROC=0.76. This study supports a strong association between US patterns and noninvasive biomarkers. There is only limited data available regarding the utility of combining bloodwork and imaging results to predict CFLD. This study highlights the potential of this combination to identify CFLD and distinguish NL, HTG, and NOD patterns [31].

It is important to determine the age and interval at which the US should be performed as a routine component of liver screening. It is unlikely to be cost-effective to perform annual abdominal US on all individuals with CF [10].

Liver stiffness measurement (Fibroscan)

The transient elastography probe uses a mechanical vibration that creates a shear wave within the liver parenchyma and is ready to read the velocity reflected from the liver surface, which shows changes according to the degree of liver fibrosis. It then gives a measurement reflecting the degree of liver stiffness, which is displayed in kilopascal (kPa) [31].

By identifying stiffness with Fibroscan in CF, Peter et al. reported that the mean stiffness in patients with CF was 5.63 ± 4.02 kPa. Liver stiffness measurements were significantly higher in the patient group with clinical [11.07 ± 5.51 kPa (n=6) vs. 5.08 ± 3.45 kPa (n=60), p<0.0001], biochemical CFLD [7.40 ± 3.10 kPa (n= 7) vs. 5.42 ± 4.08 kPa (n= 59), p=0.013] and ultrasound-identified liver disease [Williams score ≥4: 8.19 ± 5.96 kPa (n=23) vs. 4.27 ± 0.94 kPa (n= 41), p<0.0001] [3]. This study was also able to identify that genotype, age at diagnosis, age at evaluation, past medical history of meconium ileus and pancreatic insufficiency are risk factors in the development of CFLD in the studied population, which is in accordance with the current literature [3]. A more detailed comparison of the diagnostic accuracy of ultrasound and Fibroscan in the detection of CFLD in this study shows that Fibroscan is not inferior or superior to Ultrasound.

A meta-analysis was performed by Lam et al. involving 605 patients with the primary aim of determining the usefulness of transient elastography in the detection of CFLD and the secondary aim of determining the optimal cut-off. This study identified the optimal cut-off for LSM as ≥5.95 kPa, yielding a sensitivity, specificity, PPV, NPV, AUROC, and accuracy of 55%, 87%, 65%, 83%, 0.76, and 78% respectively. The optimal APRI cut-off was ≥0.329, yielding a sensitivity, specificity, PPV, NPV, AUROC, and accuracy of 52%, 93%, 66%, 88%, 0.78, and 84% respectively. When LSM with ≥5.95 and APRI with ≥0.329 combined, it yields a sensitivity, specificity, PPV, NPV, AUROC, and accuracy of 43%, 99%, 92%, 87%, and 0.87 respectively. This indicates that patients with LSM and APRI below the cut-offs are unlikely to have CFLD [32].

One examination with Fibroscan can be done under five minutes and is feasible even in young patients. It has been validated in chronic liver disease in adults, including hepatitis B, hepatitis C, primary biliary cirrhosis, and NAFLD [33]. In contrast to its use in pediatric NASH [34], the investigation was facilitated due to the pulmonary hyperinflation enlarging the intercostal space and the non-obesity of these patients. Fibroscan has proven to have excellent inter- and intra-observer, inter-site, and inter-equipment agreements and overall superiority in the detection of fibrosis when compared to ultrasound [35]. Fibroscan, when compared to other methods, is easier to learn, and does not require any professional training. It can estimate the existing degree of liver damage as well as monitor disease progression or regression via serial measurements.

Acute hepatitis (with a flare of transaminases), acute liver damage, or extrahepatic cholestasis in jaundiced patients can lead to an overestimation of the degree of liver fibrosis. Other causes for the overestimation of fibrosis include mass lesions within the liver and liver congestion. In obesity, metabolic syndrome, and ascites, unreliable readings are often identified.

Shear wave elastography (SWE)

Shear wave elastography (SWE) is a type of US elastography that uses shear waves generated by repetitive compression produced by high-intensity pulses to assess tissue elasticity and display it in a quantitative manner [36]. In SWE, the combination of a radiation force obtained through elastograms is induced in tissue by an ultrasonic beam and an ultrafast imaging sequence capable of catching the propagation of the resulting shear waves in real-time [36].

In a study to determine the usefulness of SWE, Steven et al. concluded that 6-MHz point SWE has a correlation with magnetic resonance elastography. They were also able to identify imaging criteria delineating the use of SWE to identify increased liver stiffness in children with CF. With the use of a 6-MHz point SWE of 1.45 m/s, this study was able to differentiate abnormal vs. normal with a sensitivity of 79%, and a specificity of 100% with an AUROC of 0.94. This study was also able to distinguish mild-moderate vs. severe using a cut-off of 1.84 m/s with sensitivity and specificity of 88% and 86% respectively and an AUROC of 0.79. This study also showed that APRI, FIB-4, and GPR are not as sufficient as SWE in comparison to magnetic resonance elastography [37]. This study identified a GGT cut-off that would separate children with normal vs. abnormal SWE, and a platelet cut-off that identifies children with severe liver stiffness.

Another study was able to identify a cut-off of 1.27 m/s with a sensitivity of 56.5% and specificity of 90.5% for CF liver disease [38]. Another study, which used 2.5-4 MHz transducers, identified a cut-off of 1.25 m/s for portal hypertension and 1.63 m/s for esophageal varices and end-stage cirrhosis [39].

Magnetic resonance elastography in comparison to SWE is expensive and unavailable to many patients. SWE may be helpful in filling these gaps, but there is scarce data available on how this technology should be used in patients with CF. SWE can be used as an alternative to Fibroscan in conditions like obesity and ascites when Fibroscan is not able to obtain adequate results. Also, SWE gives the operator a real-time visualization of the selected area with a large surface area compared to the Fibroscan [31].

Supersonic shear-wave elastography (SSWE)

Diego et al. studied SWE to identify liver stiffness and a combination of the results with APRI to aid in diagnostic precision. Youden's index identified 6.85 kPa as the optimal cut-point to distinguish CFLD in children with CF with a sensitivity of 75% and specificity of 71%. In this model, an LSM increase of 1 kPa detected by SSWE is associated with a 1.7-fold (95% CI: 1.3-2.4) increase in the odds of having a liver disease in pediatric CF [9]. Significant improvements were seen in ROC curve analysis combining SSWE-LSM and APRI, resulting in an improved AUC of 0.84 (sensitivity: 67%, specificity: 88%) compared to the AUC of LSM alone. SSWE combined with APRI showed a 14.8 greater odds of distinguishing liver disease in pediatric CF.

Table 1 presents a comparison of cut-off values obtained from various studies and their sensitivity and specificity. SWE has the highest AUROC, which suggests it can be used as a tool for ruling in liver disease.

**Table 1:** Comparison of cut-offs and AUROC of different tools to identify liver disease in cystic fibrosis NSD: no sufficient data; AUROC: area under the receiver operating characteristic; APRI: aspartate aminotransferase-to-platelet ratio; GPR: gamma-glutamyl transferase-to-platelet ratio; LSM: liver stiffness measurement; SWE: shear wave elastography; SSWE: supersonic shear wave elastography; HTG: heterogenous; NL: normal

Tools	Cut-offs	Sensitivity	Specificity	AUROC	P-value
APRI [12]	>0.264	73.1%	70.2%	0.75	0.0001
Fibrosis-4 [12]	NSD	NSD	NSD	0.60	0.02
GPR [5]	>0.20	74%	73%	0.81	0.004
LSM [3]	5.63 or 6.50 kPa	63%	87%	0.858	0.001
LSM + APRI [33]	≥5.95 kPa + ≥0.329	43%	99%	0.87	NSD
SWE [38]	1.45 m/s	79%	100%	0.94	NSD
SSWE [9]	6.85 kPa	75%	71%	0.79	0.0001
SWE + APRI [9]	NSD	NSD	NSD	0.84	NSD
Ultrasonography [31]	HTG vs. NL	NSD	NSD	0.76	NSD

## Conclusions

Liver disease in CF is a common entity. Early diagnosis and intervention would help plan the treatment accordingly and may increase life expectancy in these patients. There are many different screening methods available for this condition, but a minimally invasive, sensitive, and specific test is yet to be identified. Until such a test is devised, we can combine biochemical indices like APRI and GPR and imaging modalities like ultrasound, transient elastography, or SWE to maximize the strength of the diagnosis, and this concept should be included in annual guidelines. Data suggests that APRI and ultrasound are tests with high sensitivity, which can be effectively used as screening methods to rule out true negatives. Patients with positive results should undergo tests with a high specificity like GPR and transient elastography. More studies are required to validate these findings. There are plenty of variabilities to address while we are engaged in the efforts to come up with an optimal screening test, which should be kept in mind. These include age, BMI, genetic factors, pancreatic insufficiency, etc. CFLD pathology is yet to be understood, and hence so coming up with a screening test to prevent it remains the biggest challenge.
